# Case Report: A Well-Hidden Cause for Myelopathy

**DOI:** 10.3389/fneur.2021.672648

**Published:** 2021-04-20

**Authors:** Tobias Braun, Eva Schulz, Maxime Viard, Omar AlhajOmar, Tobias Struffert, Stefan Gattenloehner, Mesut Yeniguen, Martin Juenemann

**Affiliations:** ^1^Department of Neurology, University Hospital Giessen and Marburg, Giessen, Germany; ^2^Department of Internal Medicine, Agaplesion Evangelisches Krankenhaus Mittelhessen, Giessen, Germany; ^3^Department of Neuroradiology, University Hospital Giessen and Marburg, Giessen, Germany; ^4^Department of Pathology, University Hospital Giessen and Marburg, Giessen, Germany

**Keywords:** bone marrow sarcoidosis, neurosarcoidosis, myelopathy, case report, spinal cord syndrome

## Abstract

**Introduction:** Sarcoidosis is a rare, systemic inflammatory disease and can involve multiple organs, especially the lungs and lymph nodes. The nervous system is affected in <10 percent of patients, which is called neurosarcoidosis. Neurosarcoidosis can cause a multitude of symptoms and can mimic various diseases. A rare manifestation is bone marrow involvement. We describe a case of spinal cord syndrome due to myelopathy that was caused by sarcoidosis of the bone marrow.

**Case Presentation:** A male patient presented to our hospital with incomplete spinal cord syndrome. He suffered from numbness of the legs which had progressed to severe paraparesis. Magnetic resonance imaging revealed thoracic myelopathy without contrast enhancement. Thorough diagnostics found no explanation for the myelopathy, and the patient was treated symptomatically with high-dose steroids. When the patient developed non-resolving leukopenia, a bone marrow biopsy was performed. The bone marrow showed changes due to sarcoidosis. Further testing revealed myocardial involvement of the sarcoidosis. The patient was started on oral prednisolone and methotrexate. Over the course of time, his symptoms improved, but he still suffers from spastic leg paresis and needs aids to walk farther than 1 kilometre.

**Conclusion:** In patients presenting with neurological deficits of unknown cause, neurosarcoidosis is a potential explanation. If it manifests primarily in the bone marrow, the diagnosis can be easily overlooked. Abnormalities in a full blood count should make the treating physician consider this diagnosis, and a bone marrow biopsy should be performed.

## Introduction

Sarcoidosis is a rare, systemic inflammatory disease characterised by non-caseating granulomas (1). It occurs in all ages and can involve multiple organs, especially the lungs and lymph nodes (2, 3). In <10 percent of patients, the nervous system is affected, which is called neurosarcoidosis (4–6). A rare manifestation is bone marrow involvement (7). We describe the case of a young male who presented with spinal cord syndrome caused by thoracic myelopathy. It took 8 weeks to find its cause: Sarcoidosis of the bone marrow.

## Case Presentation

A male in his 40s was referred to our hospital's emergency department complaining of numbness in his lower limbs. The numbness started 3 days before in both legs and rose to the trunk over the course of time. In the days before presentation, his walking ability deteriorated, and he had problems emptying his bladder. Two days before, he perceived flu-like symptoms and shivering. Three months before, he suffered from a gastrointestinal infection. He had a past medical history of arterial hypertension and gout. His prescribed medication comprised ramipril, amlodipine, lercanidipine, and allopurinol.

The initial physical examination revealed no pareses but incomplete spinal cord syndrome with sensory loss on the level of the 9th or 10th thoracic spinal segment, slightly elevated reflexes on the left upper extremities and right lower extremities, and a distended bladder.

**Figure 1:**
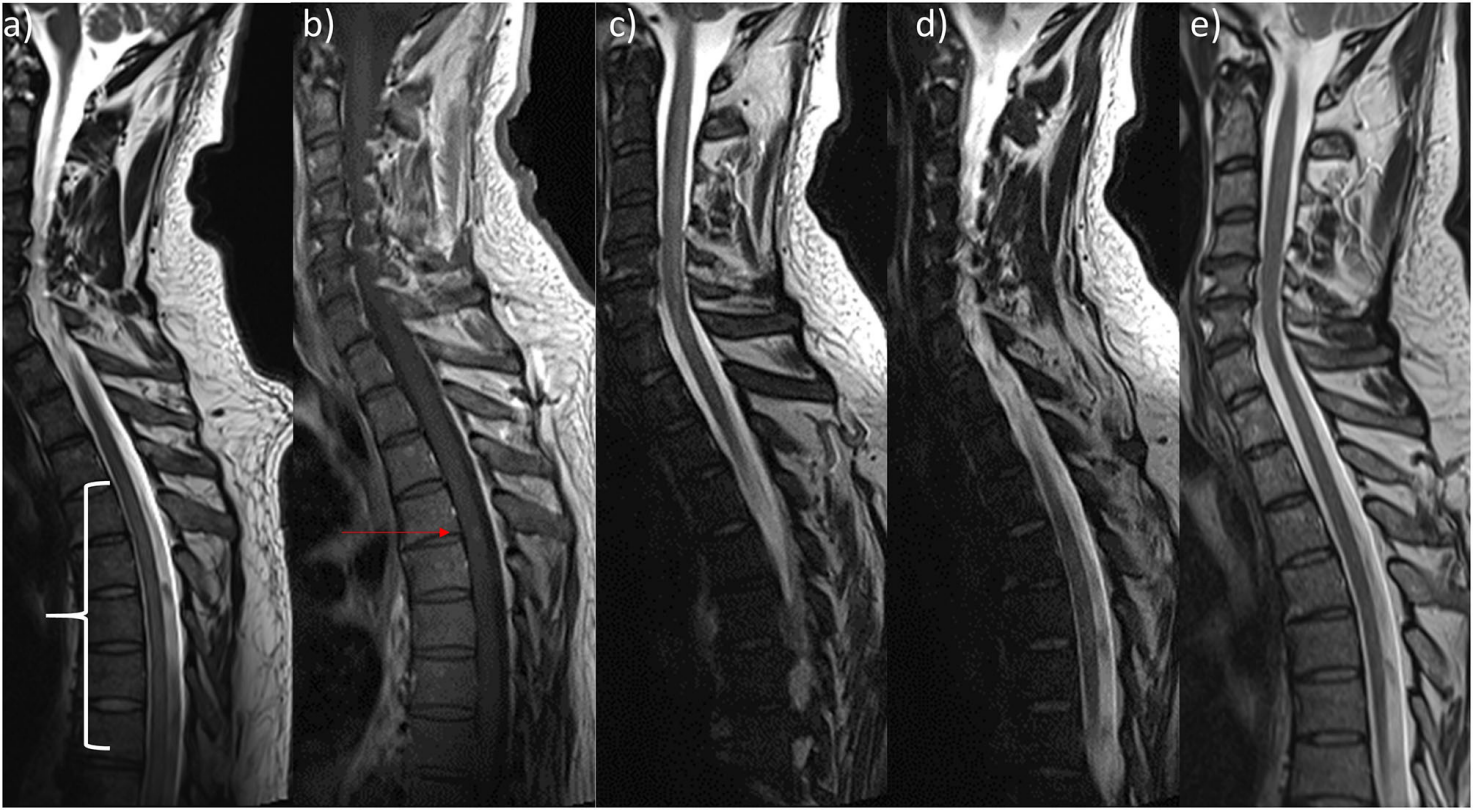
Sagittal Magnetic resonance imaging of patient's spine. **(a)** On admission there was discrete signal enhancement spanning from T4 to T11 in T2-weighted sequences. **(b)** 2 weeks later, there was a discrete contrast enhanced signal at the height of T4/5 in T1-weighted sequences. **(c,d)** Prior to discharge, T2-weighted sequences showed **(c)** a new cervical lesion spanning from C3 to C7 and the unchanged signal enhanced thoracic lesion **(d)**. **(e)** Resolvance of myelopathic changes 1 year after initial admission.

An MRI of the spine on the day of admission revealed myelopathy without contrast enhancement spanning from T4 to T11 (Figure 1a). Aortic dissection was not found on a contrast-enhanced CT-angiography scan of the thorax and abdomen. Other abnormalities were also not detected. A lumbar puncture was performed because of a suspected autoimmune or infectious cause. We found a xanthochrome cerebrospinal fluid (CSF) with pleocytosis (57/μl; normal: <5/μl) and elevated levels of protein (1.04 g/l; normal: <0.45 g/l) and lactate (2.28 mmol/l; normal: <1.9 mmol/l). Oligoclonal bands were not detected in the CSF. The patient was started on antibiotic therapy of Ceftriaxone and ampicillin and virostatic therapy of acyclovir. These therapies were stopped when microbiological and virological testing did not show any signs of bacterial or viral infections. There was no growth of bacteria in the CSF culture and viral DNA (VZV, HSV ½, EBV, CMV, Entero- and Rhinovirus) was not found using PCR. We then started the patient on a steroid pulse therapy of 1 g methylprednisolone over the course of 5 days. The symptoms slightly improved at first with this therapy, but over the next few days, the patient showed progression of the spinal cord syndrome with severe paraparesis, dysaesthesia (burning sensations), and diminished bowel function. The steroid pulse was repeated with a dosage of 2 g of methylprednisolone over the course of 5 days that id not result in any improvement of his symptoms. Therefore, the patient was treated with 7 treatment cycles of plasmapheresis that still did not lead to improvement. Due to neuropathic pain, he was started on a therapy of 200 mg of carbamazepine twice daily.

In the meantime, the patient was tested for serum-autoantibodies (ANA, ANCA, dsDNA, PR3, MPO, ß2-Glycoprotein, Cardiolipin, CCP, rheumatoid factor, mitochondrial, LKM, SLA, smooth muscles, parietal cells, AQP4, MOG) and markers for sarcoidosis (ACE, soluble IL2-receptor), which all proved negative. No evidence of HIV-infection was found. The samples were acquired prior to steroid therapy. Somatosensible and magnetic-evoked were normal from and to the upper extremities but pathologic on the lower extremities. Neuropsychological testing did not reveal any deficits but signs of reactive depression. An MRI of the brain did not show any abnormalities. Two weeks after the initial MRI of the spine, a new MRI revealed contrast enhancement on the level of T4/5 (Figure 1b).

**Figure 2:**
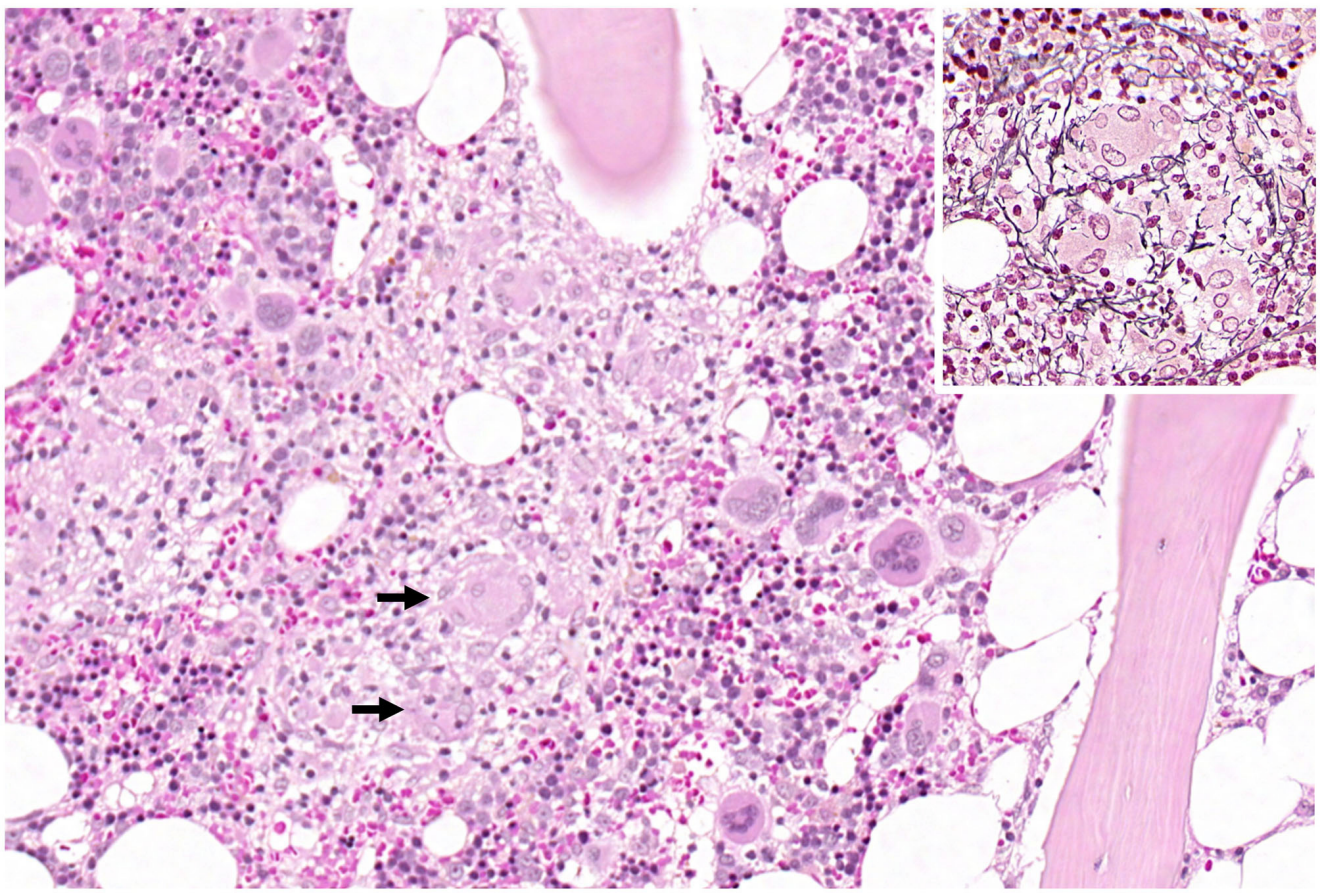
Overview of haematopoietic bone marrow with a central granuloma. The arrows mark multinucleated giant cells. The inset shows a granuloma with the typical radially arranged fibres in the reticulin fibre stain.

Several times, the patient developed elevated inflammatory markers (fever, C-reactive protein [CRP]) and was treated for pneumonia, epididymitis, and urinary tract infections with antibiotics. 4 weeks after admission, the patient developed leukopenia (3.3 10^9^/l) that further dropped over the following days and weeks (lowest: 1.2 10^9^/l) and was attributed to the carbamazepine after we discussed the case with our infectious diseases department. The patient was then tested for several bacterial infections (TBC [quantiferon test], Bartonellae, Brucellae, Coxiella) and for Hepatitis A-E. As the CRP did not resolve, transesophageal echocardiography (TEE), magnetic resonance cholangiopancreatography (MRCP) and positron emission tomography computed tomography (PET-CT) were performed. The TEE and MRCP did not provide any explanation, but the PET-CT showed bilateral central pulmonary embolization as a sign of diffuse coagulopathy, and anticoagulation was initiated. The PET-CT also showed heightened activity in the bone marrow, which was attributed to the infectious state. As the leukopenia did not resolve, the patient underwent a bone marrow biopsy. The analysis of the biopsy revealed the bone marrow was low in cells, consistent with toxic damage, myelodysplastic syndrome, or regeneration, and the patient was started on granulocyte colony stimulating factor. Leukopenia resolved 2 weeks after the first occurrence. Further testing of the bone marrow finally revealed multifocal epithelioid cell granulomas with giant cells, and 8 weeks after admission, the patient was diagnosed with sarcoidosis of the bone marrow (Figure 2). High-resolution CT of the lungs did not show any signs of pulmonary involvement, but a cardiac-MRI displayed signs of myocardial oedema, that were attributed to sarcoidosis- related inflammation. After active CMV-infection was excluded, the patient was started on 60 mg of prednisolone. Afterwards, CMV serology showed IgM-production once, which was attributed to reactivation under immunosuppression. A new MRI of the spine did show the old lesion in the thoracic myelon (Figure 1c) and a new lesion spanning from C3 to C7 (Figure 1d).

Ten weeks after initial admission, the patient was transferred to stationary rehabilitation. On discharge, the patient was able to stand with help and wiggle his toes. We recommended retesting the CMV-status and, in the case of negative CMV-status, starting the patient on a therapy of methotrexate and folate.

Five months after initial admission, the patient presented to our outpatient clinic for a routine appointment. He reported being able to walk 500 metres with help from walking aids. The bladder and bowel dysfunction had completely resolved, but he complained of weakness of the right leg and a persisting sensory deficit starting from Th9. He reported no sequelae from the pulmonary embolism, but due to the necessary anticoagulation, haematoma evacuation was necessary after a trivial trauma to the right calf. Methotrexate was started as recommended, and the prednisolone was gradually reduced to a dosage of 6 mg daily (8). On examination, there was moderate paresis of foot elevation and knee flexion and paraspasticity of the legs, with emphasis on the right side. Babinski's sign was present in the left foot. We recommended further reduction of the prednisolone, physiotherapy, and a therapeutic attempt with off-label fampridine to reduce spasticity. Fampridine was prescribed due to personal preference of the treating physician. There was no evidence-based reason.

Three months later, the patient reported being able to walk one kilometre with walking aids and walking freely at home. He still complained of weakness of the right leg and unchanged sensory deficits. A clinical examination now revealed light paresis of foot elevation and knee flexion. The remaining pre-reported symptoms were unchanged. As the fampridine did not lead to any improvement of spasticity, 5 mg of baclofen daily were recommended to reduce spasticity.

Over the course of time and 4 further appointments in our outpatient clinic, the walking distance and spasticity gradually and moderately improved. When last reporting to our outpatient clinic, the patient was able to walk 1 kilometre freely. The sensory deficits did not improve. The prednisolone was further reduced. An MRI of the spine showed complete resolution of the myelopathic changes and no contrast enhancement (Figure 1e). He had physiotherapy weekly and planned to restart his job 17 months after his first admission to our hospital.

## Discussion

In patients presenting with spinal cord syndrome, the diagnosis of myelopathy is readily made with MRI. If the origin of the myelopathy is non-traumatic, a thorough diagnostic workup is required. Angiography can identify ischemic causes. Laboratory investigations including CSF can help find hypovitaminosis or autoimmune causes (Neuromyelitis optica, multiple sclerosis, sarcoidosis, etc.). In young patients, an MRI of the brain can be performed to find cerebral evidence for multiple sclerosis.

Depending on the cause of the myelopathy, treatment can be initiated. Physiotherapy is usually necessary and can help reduce the patient's disability (9).

Sarcoidosis is a rare inflammatory disease affecting about 10/100,000 people in the USA of all ages and ethnicities (5). The non-caseating granulomas that are the hallmark of the disease contain macrophages, multinucleated giant cells, and epithelioid cells. Among others, T-cells promote the cellular immune reaction, and B-cell hyperactivity leads to immunoglobulin production (10).

A comparatively benign entity is Löfgren syndrome, an acute form of sarcoidosis with polyarthritis, erythema nodosum, and bilateral hilar adenopathy of the lung, that usually resolves without sequelae (11). In chronic sarcoidosis, the lungs can be affected, but extrapulmonary manifestations can mimic various diseases. It can affect the eyes, skin, lymph nodes (most common), heart, joints and bones, liver, spleen, and kidneys (10). If the sarcoidosis manifests in the nervous system (neurosarcoidosis), patients suffer from cranial nerve dysfunction, aseptic meningitis, headaches, focal symptoms if the brain tissue is affected, and in the case of granuloma obstructing the flow of CSF, raised intracranial pressure. Myelopathy is rare in neurosarcoidosis (4). The gold-standard for diagnosis is histopathological evaluation of the affected organs (10). In isolated neurosarcoidosis, a meningeal or brain biopsy can lead to the diagnosis (citation).

Sarcoidosis of the bone marrow is very rare. The incidence of granulomas in bone marrow biopsies is low. In these cases, up to 21 percent are attributed to sarcoidosis (5). We were unable to identify other cases of myelopathy due to bone marrow sarcoidosis.

In our patient, the non-resolving leukopenia, that was attributed to toxic bone marrow damage, led to the bone marrow biopsy. Isolated leukopenia has been described as an initial presentation of sarcoidosis secondary to bone marrow infiltration (12).

Anaemia is the most common finding in a complete blood count in bone marrow sarcoidosis (13). In our patient, the lowest level of haemoglobin was 113 g/l, which was mild and would not have led to further diagnostic testing if the leukopenia had not been present. Without these findings, we probably would have missed the diagnosis.

Immunomodulatory therapy is usually initiated in sarcoidosis, and patients are usually started on a course of prednisolone. In addition, methotrexate, azathioprine, infliximab, or rituximab are used to reduce the need for prednisolone to circumvent side effects. In neurosarcoidosis, the treatment is more aggressive, as early immunosuppression is associated with better a prognosis (14).

It should be noted as a limitation, that we should have performed conventional angiography early after the patient presented to our hospital. Thereby, an arterial or venous thrombosis, venous fistula, or venous hypertension as a cause for myelopathy might have been overlooked (9).

## Conclusion

In patients presenting with neurological deficits of unknown causes, neurosarcoidosis is a potential explanation. If it manifests primarily in the bone marrow, the diagnosis can be easily overlooked. Abnormalities in a full blood count should make the treating physician consider this diagnosis, and a bone marrow biopsy should be performed.

## Data Availability Statement

The original contributions presented in the study are included in the article/supplementary material, further inquiries can be directed to the corresponding author/s.

## Ethics Statement

Ethical review and approval was not required for the study on human participants in accordance with the local legislation and institutional requirements. The patients/participants provided their written informed consent to participate in this study. Written informed consent was obtained from the individual(s) for the publication of any potentially identifiable images or data included in this article. Written informed consent was obtained from the patient for publication of this Case report and any accompanying images. A copy of the written consent is available for review by the corresponding author.

## Author Contributions

TB, ES, MV, OA, MY, and MJ treated the patient. TS was responsible for the acquisition and interpretation of neuroradiologic imaging. SG was responsible for histological examinations and analyses. TB and MJ wrote the manuscript. All authors were involved in the analysis and interpretation of findings, they proved the manuscript, contributed for important intellectual content, and contributed to writing and approved the final manuscript.

## Conflict of Interest

The authors declare that the research was conducted in the absence of any commercial or financial relationships that could be construed as a potential conflict of interest.

## Abbreviations

CT: computed tomography
CSF: cerebrospinal fluid
ECG: electrocardiogram
MRI: magnet resonance imaging
MRCP: magnetic resonance cholangiopancreatography
PET-CT: Positron emission tomography computed tomography
TEE: transesophageal echocardiography.
